# Reducing tau acetylation rescues cognitive deficits in a mouse model of traumatic brain injury‐induced acceleration of Alzheimer’s disease

**DOI:** 10.1002/alz.091710

**Published:** 2025-01-03

**Authors:** Sarah Barker, Edwin Vázquez‐Rosa, Salvatore Caradonna, Ryan Kornblit, Aristotle Apostolakis, Lijun Dou, Min‐Kyoo Shin, Sofia Corella, Sunil Jamuna Tripathi, Suwarna Chakraborty, Kalyani Chaubey, Kathryn Franke, Coral Cintrón‐Pérez, Preethy Sridharan, Jung A Woo, David E. Kang, Li Gan, Brigid M. Wilson, Feixiong Cheng, Bindu D Paul, Andrew A. Pieper

**Affiliations:** ^1^ Louis Stokes Cleveland VA Medical Center, Cleveland, OH USA; ^2^ University Hospitals Cleveland Medical Center, Cleveland, OH USA; ^3^ Case Western Reserve University, Cleveland, OH USA; ^4^ Brown University, Providence, RI USA; ^5^ Cleveland Clinic, Cleveland, OH USA; ^6^ College of Pharmacy and Research Institute of Pharmaceutical Sciences, Seoul National University,, Seoul, Seoul Korea, Republic of (South); ^7^ Johns Hopkins University School of Medicine, Baltimore, MD USA; ^8^ Johns Hopkins University, Baltimore, MD USA; ^9^ Helen and Robert Appel Alzheimer’s Disease Research Institute, Brain and Mind Research Institute, Weill Cornell Medicine, New York, NY USA

## Abstract

**Background:**

TBI is the 3rd greatest risk factor for developing AD, behind genetics and aging. TBI is associated with a 3‐4 year earlier onset of cognitive impairment, and increased cortical thinning and amyloid plaques in people with AD. The underlying mechanisms of this relationship are not understood, and as a result there are no treatments that protect patients from accelerated AD after TBI. We reported that tau is pathologically acetylated after TBI. Acetylated tau is significantly more elevated in the brains of human AD subjects with a history of TBI, compared to AD alone and healthy controls. Therefore, we hypothesized that early tau acetylation after TBI is mechanistically involved in the acceleration of AD.

**Method:**

We developed a mouse model of TBI‐mediated acceleration of AD‐like pathology and cognitive impairment in 5xFAD mice. Our unique blast mediated multimodal TBI model is reproducible and clinically relevant. 8‐week‐old male and female 5xFAD and WT littermates underwent sham/TBI and were assessed after two weeks via Morris water maze, immunohistochemistry, western blot, and single nuclear RNA sequencing. For treatment studies, the FDA‐approved non‐steroidal inflammatory drug diflunisal, which inhibits the p300‐CBP acetyltransferase enzyme that acetylates tau, or vehicle was initiated 24 hours after TBI and continued daily. To investigate potential mechanisms, we developed an *in vitro* TBI model with Hela cells that stably express amyloid precursor protein or tau.

**Result:**

TBI causes learning deficits in young 5xFAD mice that do not occur in sham 5xFAD mice or in TBI‐injured WT littermates. TBI accelerates amyloid plaques in 5xFAD mice. 5xFAD mice show greater elevation of acetylated tau after TBI, compared to WT mice. Hippocampal snRNA sequencing showed that TBI or 5xFAD mutations alone primarily perturb neurons. By contrast, TBI in 5xFAD mice drastically alters transcription in multiple cell types. Diflunisal treatment reduces acetylated‐tau and rescues behavior deficits after TBI in 5xFAD mice. Amyloid precursor protein and tau expressing cells are more susceptible to *in vitro* TBI, compared to wild type cells.

**Conclusion:**

TBI accelerates onset of AD‐like pathologies in 5xFAD mice. Preliminary evidence shows that pharmacologically reducing acetylated tau rescues TBI‐induced cognitive deficits in 5xFAD mice.

